# A Robust Method for the Elaboration of SiO_2_-Based Colloidal Crystals as a Template for Inverse Opal Structures

**DOI:** 10.3390/s23031433

**Published:** 2023-01-28

**Authors:** Federico Fookes, Luis Polo Parada, María Fidalgo

**Affiliations:** 1 Instituto de Desarrollo Tecnológico para la Industria Química (INTEC), Universidad Nacional del Litoral–Conicet, Güemes 3450, Santa Fe 3000, Argentina; 2 Department of Civil and Environmental Engineering, University of Missouri, Columbia, MO 65211, USA; 3 Department of Medical Pharmacology & Physiology, Dalton Cardiovascular Research Center, University of Missouri, Columbia, MO 65211, USA

**Keywords:** photonic crystal, colloidal deposit, inverse opals, Bragg diffraction

## Abstract

Photonic crystals (PCs) are nanomaterials with photonic properties made up of periodically modulated dielectric materials that reflect light between a wavelength range located in the photonic band gap. Colloidal PCs (C-PC) have been proposed for several applications such as optical platforms for the formation of physical, chemical, and biological sensors based on a chromatic response to an external stimulus. In this work, a robust protocol for the elaboration of photonic crystals based on SiO_2_ particle (SP) deposition using the vertical lifting method was studied. A wide range of lifting speeds and particle suspension concentrations were investigated by evaluating the C-PC reflectance spectrum. Thinner and higher reflectance peaks were obtained with a decrease in the lifting speed and an increase in the SP concentrations up to certain values. Seven batches of twelve C-PCs employing a SP 3% suspension and a lifting speed of 0.28 µm/s were prepared to test the reproducibility of this method. Every C-PC fabricated in this assay has a wavelength peak in a range of 10 nm and a peak width lower than 90 nm. Inverse-opal polymeric films with a highly porous and interconnected morphology were obtained using the developed C-PC as a template. Overall, these results showed that reproducible colloidal crystals could be elaborated on a large scale with a simple apparatus in a short period, providing a step forward in the scale-up of the fabrication of photonic colloidal crystal and IO structures as those employed for the elaboration of photonic polymeric sensors.

## 1. Introduction

Photonic crystals (PCs) are nanomaterials made up of periodically modulated dielectric materials that exhibit a photonic band gap (PBG) [1]. Light with certain wavelengths located in the PBG is reflected; PCs can exhibit a vivid and bright appearance if the prohibition band is in the range of visible light [2,3]. Based on their geometry, these structures can be classified as one-, two-, and three-dimensional photonic crystals (3D PCs) [4]. In particular, 3D PCs are composed of materials of different refractive indexes ordered periodically in three spatial directions. Due to their distinct characteristics, PCs show great potential for developing eco-friendly printings and paintings, multiple anti-counterfeiting technologies, low-loss waveguides, optical fibers, and novel display devices, with application in several fields such as smart materials, microfluidics, telemedicine, and sensors [1,5].

In particular, owing to its simple fabrication methods, colloidal PCs (C-PC) made from monodisperse nanoparticles have been employed in coatings [6,7,8], films for surface-enhanced Raman scattering [9], chromatographic columns packing [10,11], and reflectometric interference spectroscopy [12,13,14]. Additionally, C-PCs have been studied as sacrifice templates to create inverse opals (IO) employing inorganic, organic, metallic, and ceramic materials, which are exact inverse replicas of C-PC structures and therefore maintain their optical properties [15]. Due to the uniform and well-controlled pore sizes, ordered periodicity, and interconnected pore systems, IOs have been explored for a wide range of fields including biomedical scaffolds [16], membranes [17], solar cells, photocatalysts [18], and sensors for the detection of different compound families such as explosives [19], pesticides [20,21,22], organophosphates [23,24], drugs [25,26], antibiotics [27,28], and hormones [29,30], among others [31,32].

Several manufacturing techniques have been applied to the fabrication of C-PCs that involve the self-assembly of monodisperse colloidal particles by spin and spray coating, interfacial assembly and inkjet printing, sedimentation, or vertical deposition [33]. 

Denkov et al. introduced one of the simplest methods for C-PC fabrication based on convective particle aggregation [34]; highly ordered structures were obtained due to the interplay of convective drag force, electrostatic repulsion, and capillary attraction caused by the evaporation of the solvent at the liquid meniscus in contact with the solid substrate. A successful crystal formation requires a substrate surface that is wet by the solvent, producing a low contact angle at the meniscus and a weak substrate–particle interaction, so that colloids in the interface are allowed to move and rearrange to deposit into a hexagonal array. Temperature, humidity, and colloid concentration affect the thickness of the resulting C-PC, which can range from single monolayers to several tens of micrometers. 

**Figure 1:**
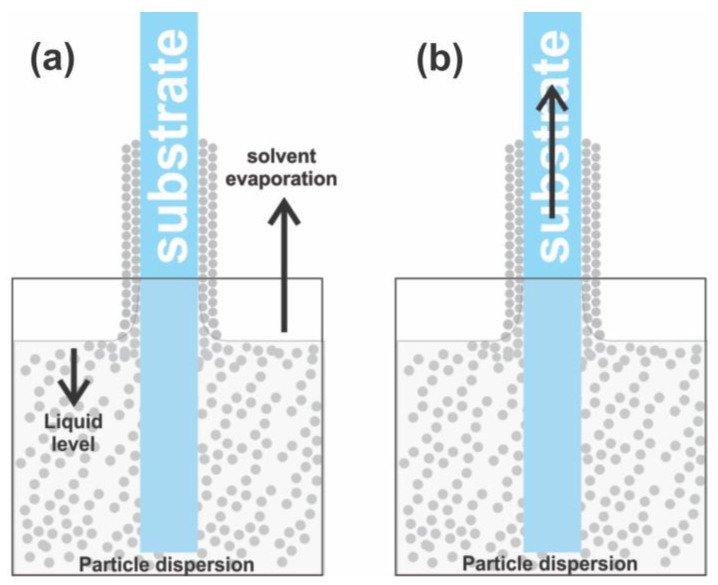
C-PC elaboration by solvent volatilization (**a**) or vertical lifting deposition method (**b**).

In practice, due to its simplicity, C-PC crystals based on convective particle aggregation are elaborated either by the method of convective assembly by solvent volatilization or vertical lifting deposition [35]. In the first case, the liquid of the particle suspension evaporates in the presence of the static substrate, while in the latter, the substrate is continuously removed at low speed from the particle dispersion (Figure 1).

Although capable of producing good quality crystals, the solvent volatilization–convective assembly method is slow and has low uniformity between deposits in terms of thickness and reflectance spectrum characteristics, most likely due to slight variations in temperature, humidity, arrangement geometry, and particle suspension properties that influence the outcome. As C-PCs find more applications in the manufacturing of advanced materials, the development of a robust methodology for C-PC fabrication that yields a large quantity of crystals with an acceptable and constant reflectance spectrum in a short period of time is critical. Despite its importance, the fabrication process and scale-up of the process have not been explored before. Currently used approaches are extremely time- and labor-intensive, limiting the scale-up of the technology, and this has therefore impaired its translation to applications and product development outside the laboratory.

In this work, a redesigned protocol for the elaboration of photonic crystals based on SiO_2_ particle deposition using the vertical lifting method is proposed and optimized for the fabrication of C-PCs. A wide range of lifting speeds and colloidal suspension concentrations were investigated. The reflectance spectra of the obtained deposits were collected, and key parameters were studied, such as maximum reflectance (% R max), wavelength at % R max, and reflectance peak full width at half maximum (FWHM). The experimental conditions that led to the best performance were selected and employed for reproducibility tests. Finally, we demonstrated the application of the C-PCs as sacrificial templates for polymeric IOs that retain the original photonic properties.

## 2. Materials and Methods

### 2.1. Materials

Monodispersed silica particles (SP) were obtained from Pinfire – Gems & Colloids (Frankfurt, Germany), while acrylic acid (AA) (99%), ethylene glycol dimethacrylate (EGDMA) (98%), ethanol (99.5%, 200 proof), 2,20-azobisisobutyronitrile (AIBN) (98%), and HF were purchased from Sigma–Aldrich (St. Louis, MO, USA). Glass and PMMA slides, both with dimensions 1 × 25 × 76 mm, were purchased from ePlastics (San Diego, CA, USA) and Fisher Scientific (Pittsburgh, PA, USA), respectively. For final use, the slides were cut using a CNC machine to obtain slides with a dimension of 1 × 5 × 76 mm.

### 2.2. Methodology

#### 2.2.1. Colloidal Crystal Elaboration

In order to obtain the C-PC, SiO_2_ particles were deposited on clean glass slides (1 × 5 × 75 mm) by vertical lifting of the support from a particle suspension at a controlled speed. Different lifting speeds (from 15 to 0.28 µm/s) and particle concentrations (from 1 to 8% wt. in ethanol) were studied.

**Figure 2:**
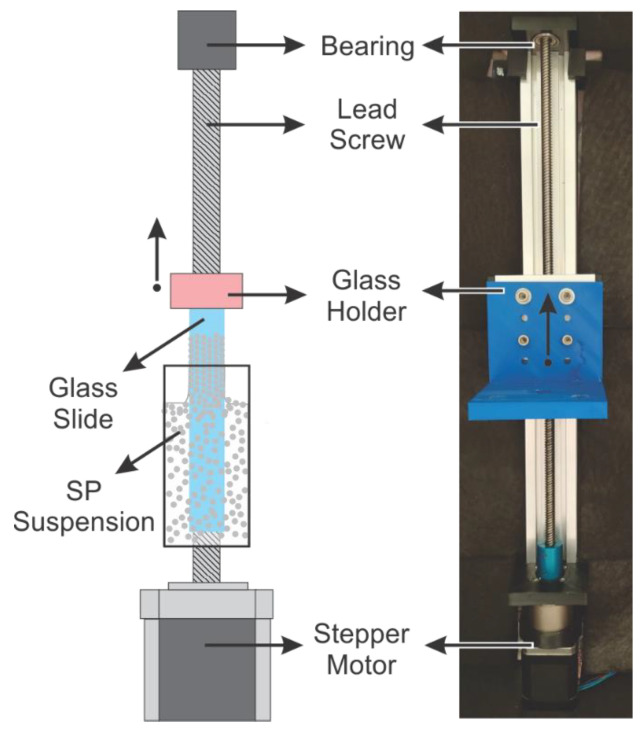
Custom-made experimental chamber employed for the C-PC fabrication.

A custom-made experimental chamber was employed (Figure 2). The machine allows for the precise control of stepper motor velocity and slide level height during the deposition. The system is provided with a stepper motor supplemented with a stepper-motor gear reduction controlled with a microcontroller (ESP-32) using a LV8729 stepper driver (Adafruit, New York, NY, USA). The final resolution of the entire system combo was 1.5625 µm/step; the lifting speed was adjusted, including a delay between steps.

Colloidal deposits were formed on both sides of the glass supports; each one had an approximate surface of 40 mm^2^. Eight different lifting speeds (from 0.28 to 15 µm/s) and SP concentrations (from 1% to 8% wt.) were assayed, for a total of 64 experimental conditions and 6 replicates at each condition. Optic parameters (maximum reflectance, peak wavelength, and FWHM), as well as the appearance of the crystals to the naked eye, were recorded to evaluate quality and reproducibility.

#### 2.2.2. Fabrication of Polymeric IOs

In order to elaborate polymeric IOs based on the C-PC, a polymerization solution composed of 439 µL of AA (monomer), 302 µL of EGDMA (crosslinker), 12 mg of AIBN (initiator), and 1 mL of ethanol (solvent) was used. A PMMA slide (1 × 5 × 75 mm) was placed on each side of the glass slide with C-PCs. This sandwich-like structure was firmly held, and then the tip was immersed in the polymerization solution in order to fill the deposit void volume with the solution. The polymerization reaction was carried out under UV (power = 36 kW, wavelength= 365 nm) for 4 h. Then, it was immersed in a 5% HF solution for 36 h to separate the polymeric films from the glass slides and to etch away the SP, and the films were rinsed with deionized water.

#### 2.2.3. Reflectance Spectrum Analysis

The reflectance spectra of the C-PCs and films were collected over a range of *λ* between 300–800 nm, using a double-beam Cary 60 UV-visible spectrophotometer (Agilent, Santa Clara, CA, USA) with an ERA-30G Specular Reflection Accessory (Harrick Scientific, Pleasantville, NY, USA) for measurement of the reflectance at a fixed angle of 30 degrees.

The diffraction wavelength of the 3D photonic crystal can be determined by Bragg–Snell’s law (Equation (1)).

(1) $$m\lambda =2n_{eff}dcos\theta$$

where λ is the diffraction wavelength, *m* is the diffraction order, *n_eff_* is the effective refractive index of the system, d is the interplanar spacing, and θ is the incident angle [36,37].

The diffraction wavelength can be also calculated using the distance between the center of the nearest spheres in (111) planes (*D*), taking into account that the colloidal particles tend to self-assemble into (111) planes of face-centered cubic (FCC) lattices in the direction parallel to the sample surface [36]. Therefore, the Bragg law can be expressed by (Equation (2)).

(2) $$m\lambda =\sqrt{\frac{8}{3}}D{\left(n_{eff}^{2}-{sin}^{2}\theta \right)}^{\frac{1}{2}}$$

The effective refractive index (*n_eff_*) can be expressed as Equation (3), where *n_p_* and *n_m_* represent the refractive indices of deposit materials (particles and air, respectively), and *V_p_* is the volume fraction occupied for the nanoparticles, equal to 0.74 in an FCC array [6].

(3) $$n_{eff}={\left(n_{p}^{2}V_{p}+n_{m}^{2}(1-V_{p})\right)}^{\frac{1}{2}}$$

#### 2.2.4. Scanning Electron Microscopy

The colloidal crystals’ morphology and particle size were studied with Environmental Scanning Electron Microscopy (ESEM) using a FEI Quanta 600 FEG (Thermo Fisher Scientific, Hillsboro, OR, USA). The deposits were attached to stubs with conductive carbon tape and sputter with an approx. 25 nm layer of platinum using a Emitech K575x sputter coater (Quorum Technologies Ltd., Ashford, Kent, UK). The mean particle diameter was determined using ImageJ software version 1.53e (National Institutes of Health, NIH, Bethesda, MD, USA).

#### 2.2.5. Data Analysis

Every obtained spectrum was processed with a R script using the Rstudio IDE [38,39], which determined a baseline at the minimum reflectance value and extracted the maximum reflectance intensity, the wavelength at maximum reflectance, and the FWHM.

## 3. Results

### 3.1. Optimization of the C-PC Elaboration Parameters

The size of SP, determined from SEM images, gave an average diameter of 266 ± 14 nm, showing a narrow size distribution. The diffraction wavelength of these SP colloidal crystals is estimated to be 547.3 nm, according to Equations (2) and (3) and the parameters provided in Table 1. Therefore, it is expected that the C-PCs developed in the current work show a reflectance peak at or close to this wavelength.

**Figure 3:**
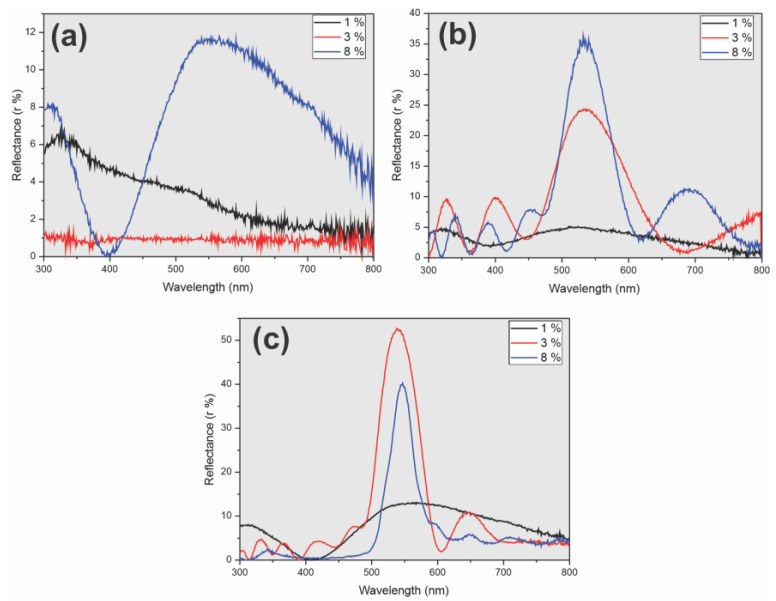
Reflectance spectra of colloidal crystals obtained at 15 (**a**), 1.66 (**b**), and 0.28 µm/s (**c**).

Reflectance spectra of deposits obtained at every assayed condition were acquired. The obtained reflectance spectra showed the studied variables, removal speed, and SP concentration have influenced the order of the colloidal crystal. At the slowest and the intermediate speed studied (0.28 and 1.66 µm/s, respectively), a marked reflectance peak was observed at about 540 nm when 3 and 8% suspensions were employed (Figure 3b,c). In contrast, this peak is diminished or disappears on deposits elaborated at the lowest SP concentrations (1%). A broad and low reflectance peak between 500 and 700 nm was obtained at the highest speed assayed when an 8% SP suspension was employed, and no peak was observed in deposits from 1 and 3% SP suspensions (Figure 3a).

**Figure 4:**
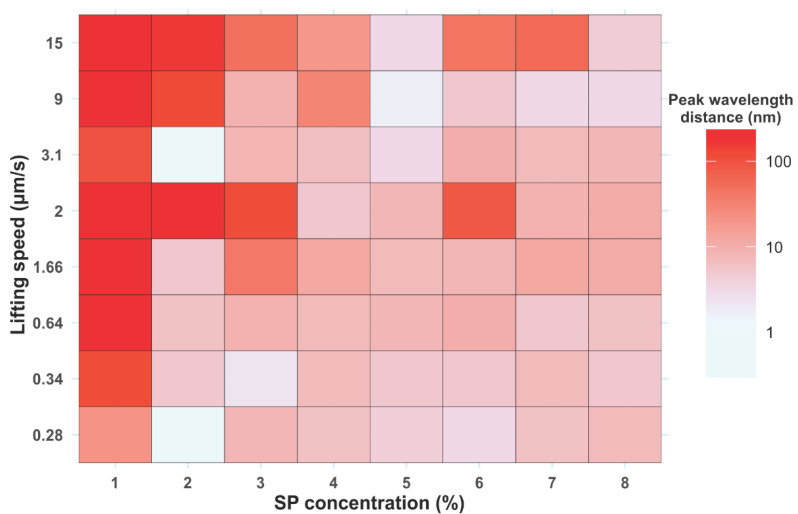
Peak wavelength distance of C-PC to the calculated value, as a function of lifting speed and particle concentration.

**Figure 5:**
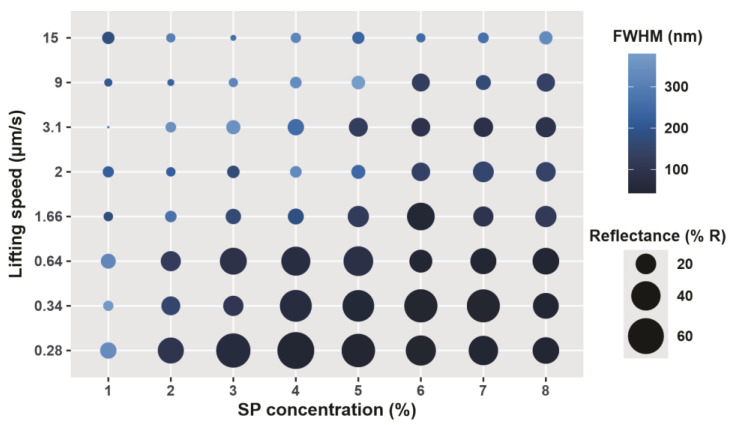
FWHM and peak reflectance intensity (R%) of C-PC, as a function of lifting speed and particle concentration.

Figure 4 and Figure 5 display a graphical representation of peak wavelength, maximum reflectance, and FWHM as a function of lifting speed and SP suspension concentration. As mentioned above, the photonic band gap is expected to be at approximately 547 nm. All colloidal crystals elaborated at speeds below 1.66 µm/s and particle concentrations greater than 1% showed good agreement with the calculated value (Figure 4, Table S1). In contrast, at higher lifting speeds and lower SP concentrations, wide and low-intensity peaks located at more than 100 nm from the estimated value were observed (Figure 4 and Figure 5, Tables S1–S3). A decrease in the lifting speed produced peaks with lower FWHM and higher intensity: colloidal crystals elaborated with a 3% SP suspension at 1.66 µm/s showed broad (FWHM 167 nm), low-intensity (12%) reflectance peaks, while those fabricated at 0.28 µm/s gave a sharper (FWHM 66 nm) and more intense (66%) one (Figure 5, Table S2). Considering that these C-PCs are highly sought as templates for photonic structures, tall, narrow peaks are desirable.

**Figure 6:**
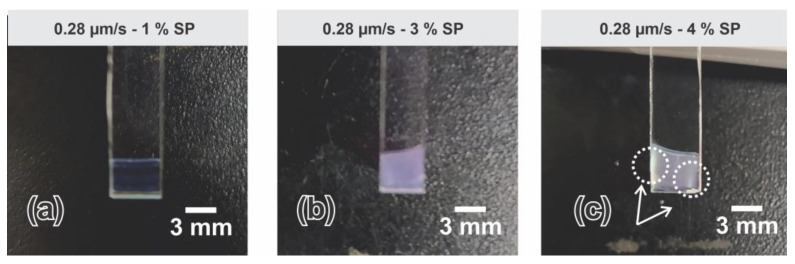
Images of C-PC fabricated at 0.28 µm/s and (**a**) 1%, (**b**) 3%, and (**c**) 4% SP concentration.

At the lowest speed considered of 0.28 µm/s, SP concentration influenced both % R and FWHM. The width of the reflectance peak decreased with increasing SP concentration until 4% and stayed somehow constant for higher concentrations. On the other hand, the % R reaches the highest value between 3 and 5%. While deposits obtained at the mentioned speed presented a homogeneous appearance in the range between 1 and 3% (Figure 6a,b), an unexpected defect was observed at higher particle concentrations tested; every deposit obtained at 0.28 µm/s and SP concentration higher than 4% presented a thick and uneven coating (Figure 6c). These anomalous regions have an opaque and non-homogeneous appearance that negatively affected the C-PC optical response.

**Figure 7:**
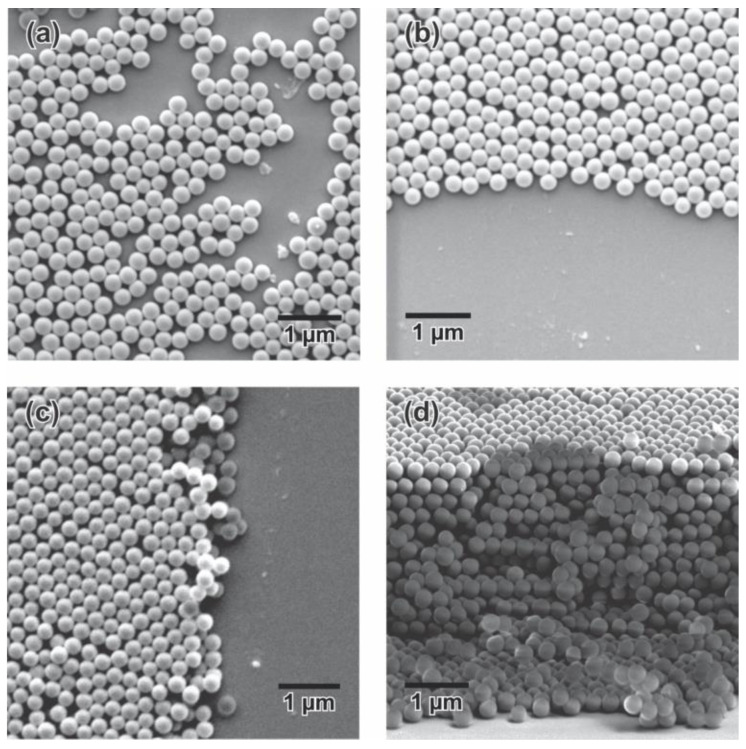
SEM micrograph of C-PCs elaborated at different lifting speeds-SP concentrations: (**a**) 2.39 µm/s^−1^ % SP, (**b**) 2.39 µm/s^−3^ % SP, (**c**) 0.28 µm/s^−1^ % SP, and (**d**) 0.28 µm/s^−3^ % SP.

SEM micrographs showed a nonuniform monolayer formed at medium speeds and low SP concentrations (Figure 7a), but an increase of SP concentrations to 3% resulted in a more uniform deposit (Figure 7b). A closed-packed C-PC was observed in the deposit elaborated at 0.28 µm/s, obtaining a larger number of particle layers at higher SP concentration (Figure 7c,d).

A theoretical model based on material flux balance in the field of colloidal fabrication was proposed by Dimitrov and Nagayama [40,41], where the number of particle layers (*k*) can be estimated with Equation (4).

(4) $$k=\frac{\beta hJ_{e}\varphi }{0.605dv(1-\varphi )}$$

where *β* is the ratio between the particle and solvent velocity (generally = 1), *h* is the meniscus height, *J_e_* is the solvent evaporation rate, *φ* is the particle volume fraction, *d* is the particles diameter, and *v* is the lifting speed. The results obtained in the current work are in agreement with this model, where higher reflectance peaks, as a result of an increase in the number of particle layers deposited [42], were achieved at higher *φ* and lower *v*.

### 3.2. Reproducibility Assays

One of the limitations of the scale-up of C-PC elaboration is the high batch-to-batch variability of the obtained colloidal crystals in terms of wavelength peak and FWHM. Parameters such as solvent evaporation rate, particle aggregation and sedimentation, and change in the particle concentration at the meniscus could modify the C-PC crystalline order and thickness and, therefore, its optical response. These parameters could incur small changes between batches due to minor variations in the assay humidity and temperature and particle concentration, among other factors. Taking into account that these structures are intended to be used in the fabrication of membranes, coatings, and sensors, the elaboration of C-PCs with a reproducible structure and optical response on a large scale is highly needed if the technologies will translate out of the laboratory. 

**Figure 8:**
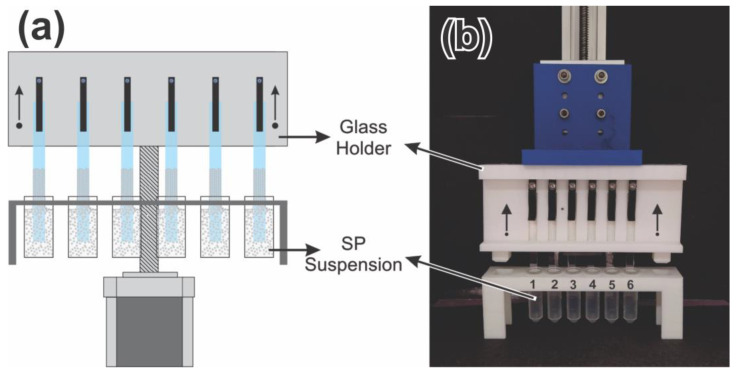
Schematic representation (**a**) and photograph (**b**) of experimental set-up employed for the reproducibility assays.

The reproducibility of the manufacturing protocol was evaluated at the optimum conditions (3% SP concentration–0.28 µm/s) for the employed experimental set-up, as determined above. Seven replicates with six glass slides each (twelve deposits) were fabricated, employing the experimental set-up displayed in Figure 8.

**Figure 9:**
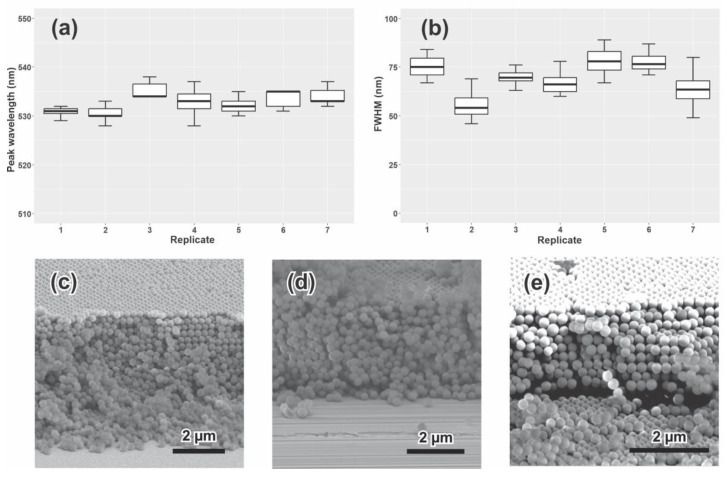
Max. reflectance (**a**) and FWHM (**b**) of batches elaborated for the reproducibility assay. SEM images of deposits obtained from three replicates (**c**), (**d**), and (**e**).

All obtained C-PCs showed the characteristic opalescent appearance with a peak wavelength between 528 and 538 nm and FWHM lower than 90 nm (Figure 9a,b). Considering that the PBG of C-PCs depends not only on the materials that form the structure (SP and air) but also on the deposit order, the similarity in the peak wavelengths suggests that the obtained C-PCs maintain a reproducible highly ordered nanostructure. Additionally, the microstructures of the C-PC obtained from different replicates (Figure 9c–e) show thick and ordered SP arrays. The methodology and conditions chosen in the current work make it possible to elaborate C-PCs on a large scale in a short period of time by employing a simple dip coating apparatus.

### 3.3. IOs and 3D Microporous Structures

**Figure 10:**
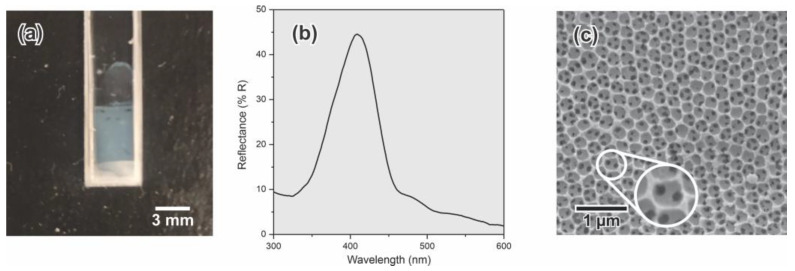
IO polymeric film: optical image (**a**), reflectance spectra (**b**), and SEM micrograph (**c**).

C-PC has been widely used as a sacrifice template for the development of 3D microporous structures and IOs. The C-PCs elaborated in the reproducibility assay were employed for the fabrication of acrylic-based IOs (such as those used, for example, for the development of photonic polymeric sensors [19,20,21,22,23,24,25,26,27,28]). The obtained materials showed a violet iridescent color (Figure 10a); their reflectance spectrum exhibited a narrow peak (74 nm) centered at 409 nm (Figure 10b). SEM images of the polymeric IOs (Figure 10c) showed a highly interconnected crystalline array of spherical porous. The internal voids of about 270 nm have a similar size to the SP used to create the deposits, and the connection between the pores are due to the particle–particle contact point. From these results, it can be concluded that the formed materials effectively replicated the C-PC nanostructure that was used as a sacrifice template for the fabrication of IOs.

## 4. Conclusions

Colloidal crystals based on monodisperse SiO_2_ particles were elaborated using the vertical lifting deposition method. In order to obtain C-PCs with appropriate photonic properties, parameters such as particle concentration and lifting speed were studied in eight different levels each by evaluating their reflectance spectrum peak wavelength and intensity. Both variables showed an effect on the selected properties. The C-PC optical response improves (lower FWHM and higher peak reflectance) with a decrease in the lifting speed and at intermediate SP concentration. The optimum results were obtained at 3% SP concentration – 0.28 µm/s. A reproducibility assay employing the optimized condition showed homogeneous and acceptable results; the wavelength peak of every replicate ranged between 528 and 538 nm with a FWHM below 90 nm. Additionally, inverse-opal films with a highly porous and interconnected morphology were elaborated using the developed C-PC as a template.

In the current work, we found the optimized conditions that make it possible to fabricate reproducible colloidal crystals that could be prepared on a large scale and in a short period of time employing a simple device. This finding provides a step forward toward a scale-up of the fabrication of C-PCs, 3D microporous structures, and IOs as those employed for the elaboration of photonic polymeric sensors.

## Figures and Tables

**Table 1:** C-PC’s physical parameters.

Particle Size (nm)	266
SiO_2_ ref. index (np)	1.46
Air ref. index (nm)	1
Effective refractive index (n_eff_)	1.35
Diffraction order (m)	1
Incident angle (θ)	30°

## Data Availability

Not applicable.

## Supplementary Materials

The following supporting information can be downloaded at: https://www.mdpi.com/article/10.3390/s23031433/s1, Table S1: Peak wavelength obtained at each experimental condition (*n* = 6, mean ± s.d.); Table S2: Maximum reflectance obtained at each experimental condition (*n* = 6, mean ± s.d.); and Table S3: Reflectance peak full width at half maximum (FWHM) obtained at each experimental condition (*n* = 6, mean ± s.d.).
