# Supplementary material for: A Robust Method for the Elaboration of SiO2-Based Colloidal Crystals as a Template for Inverse Opal Structures
Source: Sensors (Basel). 2023 Jan 28;23(3):1433. doi: 10.3390/s23031433 (PMC9920682; doi:10.3390/s23031433)
Supplement: Supplementary file 1 [file sensors-23-01433-s001.zip › sensors-2116349-supplementary.pdf]

sensors MDPI

1. Colloidal Crystals Reflectance Spectrum Analysis

The reflectance spectra of the C-PC and films were collected over a range of between 300–800 nm. The reflectance spectra of the obtained deposits were collected, and key parameters were studied, such as wavelength at maximum reflectance, maximum reflectance (% R max.), and reflectance peak full width at half maximum (FWHM), obtained results are displayed in Tables S1, S2 and S3 respectively.

Table S1. Peak Wavelength obtained at each experimental condition (n = 6, mean ± s.d.).

Peak Wavelength (nm)								
**Lifting speed**	**Nanoparticles concentration (% wt)**							
**(μm/s)**	**1**	**2**	**3**	**4**	**5**	**6**	**7**	**8**
**15**	327 ± 4	383 ± 85	598 ± 177	528 ± 12	544 ± 15	503 ± 93	606 ± 116	552 ± 11
**9**	316 ± 8	422 ± 181	538 ± 17	516 ± 4	549 ± 6	542 ± 3	544 ±2	544 ± 3
**3.1**	447 ± 98	548 ± 10	556 ± 8	554 ± 14	544 ± 7	537 ± 2	540 ± 2	539 ± 1
**2.39**	312 ± 3	352 ± 91	433 ± 130	542 ± 135	539 ± 1	540 ± 3	538 ± 1	536 ± 4
**1.66**	350 ± 79	542 ± 8	506 ± 86	535 ± 4	540 ± 5	539 ± 3	535 ± 3	536 ± 5
**0.64**	355 ± 101	541 ± 2	538 ± 3	540 ± 0	539 ± 3	537 ± 4	542 ± 3	541 ± 6
**0.34**	433 ± 195	542 ± 0	545 ± 7	540 ± 3	542 ± 1	542 ± 3	540 ± 3	542 ± 4
**0.28**	569 ± 3	547 ± 1	539 ± 2	541 ± 3	543 ± 3	544 ± 3	541 ± 5	540 ± 6

Table S2. Maximum reflectance obtained at each experimental condition (n = 6, mean ± s.d.).

Maximum reflectance (% R)								
**Lifting speed (μm/s)**	**Nanoparticles concentration (% wt)**							
	**1**	**2**	**3**	**4**	**5**	**6**	**7**	**8**
**15**	8 ± 2	6 ± 1	4 ± 2	7 ± 2	8 ± 2	6 ± 2	7 ± 4	10 ± 4
**9**	5 ± 2	5 ± 2	6 ± 2	8 ± 5	10 ± 2	16 ± 3	11 ± 2	16 ± 6
**3.1**	4 ± 1	7 ± 2	10 ± 1	13 ± 1	17 ± 5	17 ± 6	18 ± 5	19 ± 11
**2.39**	7 ± 5	6 ± 3	9 ± 3	8 ± 4	10 ± 5	17 ± 7	20 ± 13	18 ± 6
**1.66**	6 ± 2	8 ± 1	12 ± 9	13 ± 7	21 ± 18	35 ± 14	19 ± 17	21 ± 9
**0.64**	11 ± 5	19 ± 5	34 ± 5	39 ± 9	41 ± 13	24 ± 17	31 ± 17	33 ± 11
**0.34**	7 ± 4	17 ± 4	19 ± 16	47 ± 18	46 ± 28	51 ± 34	51 ± 16	26 ± 25
**0.28**	13 ± 2	32 ± 2	55 ± 12	64 ± 23	52 ± 19	42 ± 19	40 ± 17	33 ± 21

Table S3. Reflectance peak full width at half maximum (FWHM) obtained at each experimental condition (n = 6, mean ± s.d.).

FWHM (nm)								
**Lifting speed**	**Nanoparticles concentration (% wt)**							
**(μm/s)**	**1**	**2**	**3**	**4**	**5**	**6**	**7**	**8**
**15**	183 ± 37	305 ± 48	263 ± 113	320 ±34	241 ± 33	254 ± 112	267 ± 125	336 ± 60
**9**	210 ± 51	235 ± 109	319 ± 66	339 ± 99	380 ± 24	130 ± 7	173 ± 57	143 ± 24
**3.10**	297 ± 39	352 ± 61	352 ± 94	255 ± 128	128 ± 14	96 ± 5	87 ± 18	93 ± 17
**2.39**	229 ± 157	225 ± 178	170 ± 139	334 ± 65	249 ± 77	143 ± 92	157 ± 98	147 ± 80
**1.66**	179 ± 87	270 ± 72	167 ± 92	188 ± 110	123 ± 138	51 ± 7	102 ± 69	120 ± 71
**0.64**	328 ± 146	127 ± 7	91 ± 6	74 ± 9	85 ± 69	47 ± 4	47 ± 9	48 ± 17
**0.34**	375 ± 158	157 ± 53	110 ± 24	71 ± 9	56 ± 8	43 ± 7	43 ± 9	103 ± 137
**0.28**	342 ± 94	105 ± 17	66 ± 2	49 ± 6	46 ± 10	44 ± 12	48 ± 8	49 ± 7
